# Evidence against vs. in favour of a null hypothesis

**DOI:** 10.1007/s40037-017-0332-6

**Published:** 2017-02-16

**Authors:** Jimmie Leppink, Patricia O’Sullivan, Kal Winston

**Affiliations:** 1 Maastricht University, Maastricht, The Netherlands; 2 University of California, San Francisco, USA; 3 The Commonwealth Medical College, Scranton, PA USA

The overall purpose of the ‘Statistical Points and Pitfalls’ series is to help readers and researchers alike increase awareness of how to use statistics and why/how we fall into inappropriate choices or interpretations. We hope to help readers understand common misconceptions and give clear guidance on how to avoid common pitfalls by offering simple tips to improve your reporting of quantitative research findings. Each entry discusses a commonly encountered inappropriate practice and alternatives from a pragmatic perspective with minimal mathematics involved. We encourage readers to share comments on or suggestions for this section on Twitter, using the hashtag: #mededstats.

There is a widespread habit in educational research of interpreting statistically non-significant findings, also called *null findings*, as evidence in favour of a null hypothesis (i. e., ‘no difference’, ‘no relation’ or ‘no effect’). Null findings are frequently interpreted as ‘informing’ theory or as ‘confirming’ theoretical expectations. In this entry, we explain two arguments against the habit of interpreting a null finding as evidence in favour of a null hypothesis. Based on these arguments, we explain that statistical power and required sample size calculations along with replication research and meta-analysis can help us counter the habit of interpreting non-significant findings as evidence in favour of the null hypothesis, and that Bayesian hypothesis testing can help researchers to evaluate the strength of evidence in favour of the null hypothesis or against it.

## Example study

One area of study in educational research compares learning from examples with learning by solving problems [1, 2]. Suppose that a group of researchers randomly assigns 40 bachelor students in medicine to a problem-problem (*n* = 20) and an example-problem (*n* = 20) condition. Students in the problem-problem condition solve two problems – problem A and problem B – that follow the same structure and are of similar difficulty. In the example-problem condition, students first study a worked example of problem A and then solve problem B. Subsequently, students in both conditions complete the same post-test, which comprises ten problems of the same structure as problem A and B and are of similar difficulty. Each post-test problem is scored ‘0’ whenever a student provides an incorrect solution and ‘1’ when that student provides a correct solution. Hence, the total score on the post-test can range from 0 to 10.

The researchers compute post-test scores accordingly for each student and find that the two conditions do not differ much in post-test score: the problem-problem condition yields an average score of 5.775 with a standard deviation (*SD*) of 1.16, while the example-problem condition yields an average score of 6.05 with an *SD* of 0.89. The 95% confidence interval of the difference between average scores (6.05–5.75 = 0.30) [3] extends from −0.36 to 0.96 and thus includes ‘0’, meaning the null hypothesis of ‘no difference’ cannot be rejected [4]. Researchers who tend to compute a *p*-value instead of a confidence interval do a *t*-test on the difference between the average scores of the two conditions with the null hypothesis of ‘no difference’ against the alternative hypothesis of ‘there is a difference’ [5] and find: *p* = 0.36.

In many cases, the researchers use the *p*-value of 0.36 – or the 95% confidence interval that includes ‘0’ for that matter – to conclude that there is ‘no difference’ between the two conditions and hence it does not matter whether in a practical situation we let students solve problems by themselves right away or we first provide them with an example. Two main arguments against this ‘confirming the null’ approach are discussed in the following.

## Study has limited statistical power rather than evidence in favour of the null hypothesis

A first argument against interpreting non-significant *p*-values as evidence in favour of the null hypothesis comes from scholars who note that studies with sample sizes that are common practice in psychology and education (e. g. our example study) often lack statistical power and may therefore frequently fail to reject the null hypothesis even if it is not true (i. e. Type II error) [3–7]. Statistical power is the probability of being able to reject the null hypothesis if the null hypothesis is not true. Research in psychology and education should strive for a statistical power of around 0.80 [8, 9] ; with that statistical power, a statistical significance test on an untrue null hypothesis would result in a rejection of that null hypothesis in 80% of the cases [7]. Some readers might wonder why not strive for a power that lies closer to 100%; the reason for this is that many phenomena of interest in the field of education are of such a size that we would often need samples of hundreds of participants and more to achieve such a statistical power and that is ethically and logistically not always feasible.

Had the researchers of our example study, prior to conducting the study, calculated the statistical power (e. g. using G*Power [10]) for a study with two groups of *n* = 20, taking as a starting assumption for their calculation half a standard deviation difference in the population of interest and testing at the conventional *α* = 0.05 significance level, they would have learned that their study has a statistical power of only 0.34. In other words, even if there is such a difference in the population they sampled their students from, only about one of every three studies of this size (two groups of *n* = 20) would reject the null hypothesis of ‘no difference’ (i. e. find *p* smaller than 0.05). This is the same as saying that we would fail to reject the null hypothesis in about two of every three studies of this size.

Had the researchers calculated in advance what sample size they would need for a statistical power of 0.80, assuming half a standard deviation difference in the population of interest and testing at the conventional *α* = 0.05 significance level (i. e. required sample size calculation), they would have learned that they need two groups of *n* = 64 each [7].

Thus, while the researchers in our example study interpret a non-significant *p*-value as evidence in favour of the null hypothesis, a study with two groups of *n* = 20 is unlikely to detect a substantial difference between groups in the first place.

## The likelihood of a finding under competing hypotheses

A second argument against interpreting non-significant *p*-values as evidence in favour of the null hypothesis comes from scholars who point at the fact that a statistical significance test uses the *p*-value as a probability under the null hypothesis but disregards such a probability under the alternative hypothesis [11]. Scholars who use this argument state that for obtaining either evidence in favour or against the null hypothesis researchers must compare the likelihood of their finding under the null hypothesis of ‘no difference’ and the likelihood of their finding under the alternative hypothesis of ‘there is a difference’ to determine under which of these two hypotheses the finding is more likely to have occurred. The resulting *likelihood ratio* or *Bayes factor* then expresses under which of the two hypotheses – null or alternative – the observed finding is more likely to have occurred [11]:

$$\text{Bayes factor for alternative vs}.\, \text{the null}=\frac{\text{likelihood of observed finding under alternative}}{\text{likelihood of observed finding under the null}}$$

and

$$\text{Bayes factor for the null vs}.\, \text{alternative}=\frac{\text{likelihood of observed finding under the null}}{\text{likelihood of observed finding under alternative}}$$

hence:

$$\text{Bayes factor for the null vs}.\, \text{alternative}=\frac{1}{\text{Bayes factor for alternative vs}.\, \text{the null}}$$

A Bayes factor of 1 would indicate that the observed finding is equally likely under both hypotheses (i. e. numerator and denominator of the ratio are equal). A Bayes factor for the alternative hypothesis (numerator) vs. the null hypothesis (denominator) of 2 corresponds with a Bayes factor for the null hypothesis (numerator) vs. the alternative hypothesis (denominator) of 0.5 (i. e. 1/2 = 0.5) and indicates that the finding is twice as likely to have occurred under the alternative hypothesis. Analogously, a Bayes factor for the alternative hypothesis vs. the null hypothesis of 0.5 is the same as a Bayes factor for the null hypothesis vs. the alternative hypothesis of 2 (i. e. 1/0.5 = 2) and indicates that the finding is twice as likely to have occurred under the null hypothesis. Such an interpretation is impossible to achieve with a *p*-value. Table 1 provides a brief overview of the meaning of a Bayes factor in terms of evidential strength [12].

**Table 1:** Bayes factors and strength of evidence for the alternative hypothesis (numerator) vs. the null hypothesis (denominator)

Bayes factor	Evidential strength
>100 32–100 10–32 3.2–10 1–3.2	Decisive Very strong Strong Substantial Not worth more than a bare mention

For more details on the use and interpretation of Bayes factors, we refer to Rouder et al. [11], who provide a worked example of a Bayesian *t*-test as an alternative to the *t*-test that we have been using in medical education research for decades.

There is a free SPSS-like software program that enables researchers to calculate *both p*-values *and* Bayes factors [13]. Using this software program in the example study – where researchers find a difference between average scores of 0.30 – yields a Bayes factor of 2.32 for the null hypothesis of ‘no difference’ vs. the alternative hypothesis of ‘there is a difference’ (or 1/2.32 ≈ 0.43 for the alternative hypothesis vs. the null hypothesis). In other words, the finding of a difference between average scores of 0.30 is more than twice as likely to have occurred under the null hypothesis. Note, however, that this Bayes factor of 2.32 still only provides evidence (here: in favour of the null hypothesis) that is barely worth a mention (i. e. Table 1). In other words, this Bayes factor indicates some but not much preference towards the null hypothesis.

## A summary of the arguments

Following the argument of limited statistical power, calculations of statistical power and sample size to achieve a high statistical power can help us reduce the likelihood of planning a study that is too small to have a decent statistical power and can help us counter the habit of interpreting non-significant *p*-values in terms of evidence in favour of the null hypothesis. Moreover, the researchers from our example study could have a look at other studies that have also made problem-problem comparisons [1, 2] and would then notice that these other studies found rather pronounced differences.

However, in the light of the argument that we ought to compare the likelihood of a finding under the null vs. under the alternative hypothesis, we should not interpret a non-significant *p*-value as evidence in favour of the null hypothesis regardless of statistical power or sample size [11]. If we are really interested in the question how strong the evidence is in favour of a null hypothesis, we need a hypothesis testing approach that allows for comparing the likelihood of a finding under the null hypothesis with that under an alternative hypothesis, and Bayes factors can assist in that endeavour. Moreover, replication studies [14, 15] and meta-analysis [16] can help us compare findings from similar studies on the same phenomenon and, contrary to *p*-values, Bayes factors of single studies can easily be combined in meta-analysis to provide a more accurate picture across studies with regard to the evidence in favour of or against the null hypothesis [11, 12].

## To conclude

Absence of evidence is not the same as evidence of absence; *p*-values and confidence intervals may provide some evidence against a null hypothesis, but cannot provide evidence in favour of a null hypothesis. With statistical power and required sample size calculations as well as replication research and meta-analysis, we have powerful tools for countering the habit of interpreting non-significant *p*-values as evidence in favour of a null hypothesis. Besides, Bayesian hypothesis tests provide researchers with a tool to address the question of evidence in favour of the null hypothesis when that question is of interest.
